# Predicting risks of low birth weight in Bangladesh with machine learning

**DOI:** 10.1371/journal.pone.0267190

**Published:** 2022-05-26

**Authors:** S. M. Ashikul Islam Pollob, Md. Menhazul Abedin, Md. Touhidul Islam, Md. Merajul Islam, Md. Maniruzzaman

**Affiliations:** 1 Statistics Discipline, Khulna University, Khulna, Bangladesh; 2 Department of Statistics, Jatiya Kabi Kazi Nazrul Islam University, Mymensingh, Bangladesh; University of Southern Queensland, AUSTRALIA

## Abstract

**Background and objective:**

Low birth weight is one of the primary causes of child mortality and several diseases of future life in developing countries, especially in Southern Asia. The main objective of this study is to determine the risk factors of low birth weight and predict low birth weight babies based on machine learning algorithms.

**Materials and methods:**

Low birth weight data has been taken from the Bangladesh Demographic and Health Survey, 2017–18, which had 2351 respondents. The risk factors associated with low birth weight were investigated using binary logistic regression. Two machine learning-based classifiers (logistic regression and decision tree) were adopted to characterize and predict low birth weight. The model performances were evaluated by accuracy, sensitivity, specificity, positive predictive value, negative predictive value, and area under the curve.

**Results:**

The average percentage of low birth weight in Bangladesh was 16.2%. The respondent’s region, education, wealth index, height, twin child, and alive child were statistically significant risk factors for low birth weight babies. The logistic regression-based classifier performed 87.6% accuracy and 0.59 area under the curve for holdout (90:10) cross-validation, whereas the decision tree performed 85.4% accuracy and 0.55 area under the curve.

**Conclusions:**

Logistic regression-based classifier provided the most accurate classification of low birth weight babies and has the highest accuracy. This study’s findings indicate the necessity for an efficient, cost-effective, and integrated complementary approach to reduce and correctly predict low birth weight babies in Bangladesh.

## Introduction

Low birth weight (LBW) is a leading concern for public health in many developing countries, including Bangladesh. LBW refers to babies born weighing less than 2.5 kilograms [1–4]. LBW is most often caused by being born before 37 weeks of pregnancy. Being born before the 37^th^ week of pregnancy is the leading cause of LBW. Globally, more than 80% of newborn babies die due to LBW [5–8]. Around 20% of all births are estimated to be LBW globally, resulting in over 20 million births per year [4]. LBW is more than double as typical in developing countries (7%) [9]. Southern Asia is home to nearly half of the world’s LBW newborns [10]. The prevalence of LBW in Bangladesh was 17.7% in 2011 [11], 20% in 2014 [12], and 16% in 2017 [13]. That showed that LBW increased from 2011 to 2014 by 2.3% but decreased from 2014 to 2017 by 4%. Though the LBW rate has decreased in Bangladesh between 2011 and 2017, it is still higher than in most developing countries [1].

LBW was the leading cause of neonatal and under-five mortality [12]. Understanding the causes of and circumstances of LBW neonatal and under-five death is necessary to achieve the Sustainable Development Goal (SDG) 3, target 3.2, which aims to reduce neonatal mortality at 12 per 1,000 live births and under-5 mortality at 25 per 1,000 live births by 2030 [14]. However, various studies have found that LBW has a significant impact on neonatal and under-five mortality in countries such as Bangladesh [15], India [16], and Nigeria [17]. In order to achieve SDG 3, target 3.2, we need to identify the high-risk factors for LBW and take the necessary steps to reduce LBW. If we are enabled to reduce LBW, as a result, neonatal and under-five mortality will also be reduced. Brain injury, chronic lung and liver disease, deafness, blindness, epilepsy, intellectual impairment, cerebral palsy, mental retardation, developmental conditions, physical disability, cardiovascular disease, stomach complications, elevated blood pressure, type-2 diabetes, and attention deficit disorder are also risk factors for the future life of LBW babies [13, 18–20]. Both biological and demographic factors affect birth weight in Bangladesh [21]. Different demographic factors such as region, mother’s education, wealth index, cesarian delivery, delivery place, antenatal care (ANC) visit, parity, and biological factors such as mother’s height, weight, body mass index (BMI), age, twin baby, alive babies were significantly associated with LBW [13, 18–26]. The LBW rate has decreased over the last six years, but any factor of LBW’s independence remains contentious [21]. So, it is a vital issue to analyze the current factors with existing data. It will aid in determining the currently associated factors causing LBW in Bangladesh. Early ascertainment of the prominent risk factors was significantly associated with LBW, and prediction of LBW is an essential task to reduce the number of LBW babies. Previous studies were conducted in Bangladesh to ascertain the significant risk factors of LBW in Bangladesh using logistic regression (LR)-based on Bangladesh demographic and health survey (BDHS) data [15–27]. But they failed to predict LBW using machine learning (ML)-based classification techniques [15–27]. Thus, the present study aimed to determine the associated risk factors of LBW in Bangladesh using LR and predict LBW using two popular ML-based techniques, namely LR and decision tree (DT).

## Literature review

Some important literature related to identifying the most informative risk factors and predicting LBW using various ML algorithms. Eliyati et al. [28] used the Indonesia Demographic Health and Survey (DHS) 2012 dataset with 12055 respondents having eight factors of LBW. They did not use any feature selection methods to identify the high-risk factors of LBW. They took 80% dataset for the training set and 20% dataset for the test set. They used two classifiers, namely LR and support vector machine (SVM), with four kernels: Gaussian radial basis (GRB), polynomial (Poly), linear, and hyperbolic tangent (HT) for predicting LBW and found that LR achieved a higher AUC of 0.56. Senthilkumar and Paulraj [29] used the maternal details of 189 respondents, 59 of whom had LBW babies. They used different ML algorithms such as LR, naive Bayes (NB), random forest (RF), SVM, neural network (NN), and classification tree (CT) to predict LBW. CT achieved the highest accuracy of 89.9%. Hange et al. [30] worked with North Carolina State Centre for Health Statistics-2006 data with 10,000 respondents and 131 variables. For predicting LBW, they used a synthetic minority oversampling technique with three ML-based classifiers, including J48, random tree (RT), and REP Tree. They found that J48 gave 0.90 AUC. Borson et al. [31] used the BDHS-2011 and 2014 datasets, each having 4498 respondents and eight predictors. They adopted 10-fold cross-validation (CV) and six classifiers such as LR, NB, k-nearest neighborhood (k-NN), RF, SVM, and multilayer perceptron (MLP) to predict LBW. Among them, LR gained a higher AUC of 0.83.

## Materials and methods

### Sampling method and sample size

The LBW data was extracted from the Bangladesh Demographic and Health Survey (BDHS), conducted in 2017–18, and is freely accessible online [13]. BDHS used a two-stage stratified cluster sampling as a sampling method. In the 1^st^ stage of sampling, 675 enumeration areas (EAs) were chosen, whereas 250 EAs were from urban areas and 425 from rural areas, with a probability proportional to the EA scale. A complete household listing procedure was carried out in all selected EAs to provide a sampling frame for the 2nd stage sorting of households. A systematic sample of 30 homes per EA was chosen in the 2^nd^ stage of sampling to provide statistically accurate estimates of key demographic and health variables for the nation as a whole, rural and urban areas separately, and each of the eight divisions. In data collection, a total of 20,250 residential households were chosen under this plan. Face-to-face interviews using questionnaires were used to obtain data for the survey. Around 20,100 ever-married women aged 15–49 were expected to complete the interviews [13]. Each respondent was requested to provide an entire birth history for births during the survey period and take birth weight in grams. Sample weights were employed in the dataset to guarantee that the survey findings were accurately represented at the national and division levels. Excluding extreme observations and missing values and implementing weighted variables, a total of 2351 observations were selected for the final analysis.

### Ethical approval

This study analyzed an existing public domain survey dataset that was freely available online with all identifier information removed. The ethics committee in Bangladesh approved the survey. The authors were permitted to use the data for independent research purposes.

### Outcome variable

The main outcome variable of the present study was birth weight, which was measured in grams. LBW refers to a weight less than 2500 gm at birth, and Normal birth weight (NBW) refers to a weight greater than or equal to 2500 gm. For our study purpose, we defined the outcome variable as “1” for LBW and “0” for NBW [13].

### Predictor variables

In the current study, we considered several predictors based on the relevant previous works [12, 16, 20, 21, 26, 27, 32]. These included mother’s age, type of place, region, education, wealth index, marriage to 1^st^ birth interval, parity, height, weight, child is twin, child’s sex, child is alive, ANC visit, delivery place, delivery by cesarean section (CS), taking pills, and body mass index (BMI). The predictor names, predictor types, predictor descriptions, along with their categorizations, were presented in Table 1.

**Table 1:** Predictor names, predictor types, predictor descriptions, along with their categorizations.

Variables names	Variable type	Descriptions	Categorization
Mother’s age	Continuous	Mother’s age in years	--
Type of place	Nominal	Type of place of residence	(i) Urban and (ii) Rural.
Region	Nominal	The administrative region where respondents reside	(i) Barisal, (ii) Chittagong, (iii) Dhaka, (iv) Khulna, (v) Mymensingh, (vi) Rajshahi, (vii) Rangpur, and (viii) Sylhet.
Education	Ordinal	Mother’s education	(i) No education, (ii) Primary, (iii) Secondary, and (iv) Higher.
Wealth index	Ordinal	Respondents wealth index status	(i) Poorest, (ii) Poorer, (iii) Middle, (iv) Richer, and (v) Richest.
Marriage to 1^st^ BI	Nominal	Marriage to 1^st^ birth interval	(i) ≤24, (ii) 25–48, and (iii) >48
Parity	Nominal	Parity at sterilization	(i) <5 and (ii) ≥5
Height (m)	Continuous	Mother’s height in meter	--
Weight (kg)	Continuous	Mother’s weight in kilogram	--
Child is twin	Nominal	Single or multiple births	(i)Single, (ii) 1^st^ of multiple, (iii) 2^nd^ of multiple.
Child’s sex	Nominal	Sex of the child	(i) Male and (ii) Female
Child is alive	Nominal	The child is alive	(i) Yes and (ii) No
ANC visit, ≥8	Nominal	Numbers of antenatal visits	(i) <8 and (ii) ≥8
Delivery place	Nominal	Place of delivery	(i) Home, (ii) Hospital, and (iii) Others.
Delivery by CS	Nominal	Delivery in cesarean section	(i) Yes and (ii) No
Taking pills	Nominal	Taking pills in pregnancy	(i) Yes and (ii) No
BMI (kg/m^2^)	Nomial	Body mass index	(i) Underweight, (ii) Normal, (iii) Overweight, and (iv) Obesity.

BI: Birth interval

### Statistical analysis

Data for nominal and ordinal variables were expressed as a percentage (%), whereas data for continuous variables was mean±SD. We employed a chi-square test for nominal variables and an independent paired t-test for continuous variables to examine the association between different factors and LBW. A p-value<0.05 determines the statistical significance. The significant predictors were used in the LR model to determine the prominent risk factors of LBW. This work utilized Stata version 14.2 for statistical analysis and R studio version 1.3 for ML-based classifiers.

### Classifier types

To classify and predict LBW, two well-known ML-based classifiers, namely LR and DT, were adopted. A brief description of each of these classifiers is as follows.

#### Logistic regression

Logistic regression is a category of generalized linear modeling known as a logistic model or a logit model. It is a well-known and simplest supervised ML-based classification technique. This technique is widely applicable in regression and various classification problems like diabetes, LBW, hypertension, and so on [32–35]. It predicts binary outcome variables using a set of predictor variables of discrete or continuous type. Let us suppose that y is the outcome variable with membership class level (LBW/ NBW) and X = (x_1_, x_2_, …, x_6_) is the set of predictor variables. The mathematical form of the logistic regression model is

$$\text{logit}\left(\pi \right)=\text{ln}\left(\frac{\pi }{1-\pi }\right)=\alpha +\beta_{1}{\text{x}}_{1}+\beta_{2}{\text{x}}_{2}+,\ldots ,+\beta_{6}{\text{x}}_{6}$$ (1)

Equivalently, after taking exponentiation, both sides

$$\frac{\pi }{1-\pi }={\text{e}}^{\alpha +\beta_{1}{\text{x}}_{1}+\beta_{2}{\text{x}}_{2}+,\ldots ,+\beta_{6}{\text{x}}_{6}}$$ (2)

Where, π indicates the probability of occurring event LWB and 1-π indicates the probability of ocurring event NBW and α, β_1_, β_2_,…,β_6_ be the parameters. The parameters are estimated by using the maximum likelihood estimation method.

#### Decision tree

Decision tree (DT) is one of the first tree-based supervised ML techniques [36]. The core objective of DT is to create a training model for predicting membership class level (LBW/NBW) by lowering the generalization error [37]. To construct a model, DT contains multiple levels, in which the top-most node is usually called the root node, every internal node (child node) denotes a test on an input predictor variables or factors, every branch denotes the outcome of the test set. Every leaf/terminal node denotes the membership class label. It can handle both categorical and continuous data. It requires minimum data preparation and can analyze massive datasets quickly.

### Holdout cross-validation and performance evaluations

A holdout approach is a simple form of cross-validation (CV) in which the dataset is partitioned into two sets as a training set, and a test set. In this present work, we split the dataset at the following ratios: 70:30, 75:25, 80:20, 90:10. We fit the predictive model for the training set and predict the class (LBW vs. NBW) based on the test set. The predictive power of the models were measured using accuracy, sensitivity (Se), specificity (Sp), positive predictive value (PPV), negative predictive value (NPV), and area under the curve (AUC) [38, 39].

## Results

### Baseline and demographic characteristics of the respondents

The baseline and demographic characteristics of the participants are shown in Table 2. The average prevalence of LBW in Bangladesh was 16.2%. The average age of a mother whose baby with LBW was 24.8±5.9, with a height of 1.5±0.1 and a weight of 51.49±10.9. About 15.6% of LBW babies came from the Dhaka region. It was noted that 14.8% of LBW babies were delivered by cesarean section (CS). Table 2 indicates that region, education, wealth index, weight, height, twin child, child alive, and delivery by CS were statistically significantly associated with LBW.

**Table 2:** Baseline and demographic characteristics of LBW babies.

Factors	Total	LBW	NBW	p-value^1^
Total, n (%)	2351	381 (16.2)	1971 (83.8)	**—**
Age	24.8±5.5	24.8±5.9	24.8±5.4	0.732
Type of place, Rural, n (%)	1551(66.0)	250 (16.1)	1,301 (83.9)	0.919
Region, Dhaka, n (%)	713 (30.3)	111 (15.6)	602 (84.4)	0.006
Education, Secondary, n (%)	1229 (52.3)	192 (15.6)	1037 (84.4)	<0.001
Wealth, Richest, n (%)	727 (30.9)	97 (13.3)	630 (86.7)	0.019
Marriage to 1^st^ birth interval	27.1±25	26.8±24.7	24.7±25.1	0.730
Parity, <5, n (%)	85 (3.6)	11 (13.2)	74 (86.8)	0.375
Height (m)	1.5±0.1	1.5±0.1	1.5±0.1	<0.001
Weight (Kg)	53.4±10.9	51.49±10.9	53.6±10.8	<0.001
Twin child, Single, n (%)	2297 (97.7)	338 (14.7)	1959 (85.3)	<0.001
Sex, Male, n (%)	1262 (53.7)	187 (14.8)	1075 (85.2)	0.098
Child is alive, No, n (%)	71 (3.0)	28 (39.3)	43 (60.7)	<0.001
ANC visits, ≥8, n (%)	394 (16.8)	58 (14.7)	336 (85.3)	0.755
Delivery, Hospital, n (%)	2104 (89.5)	335 (15.9)	1769 (84.1)	0.374
Delivery in CS, Yes, n (%)	1455 (61.9)	216 (14.8)	1239 (85.2)	0.029
Iron pills, Yes, n (%)	173 (7.4)	30 (17.2)	143 (82.6)	0.441
BMI, Overweight, n (%)	550 (23.4)	89 (16.2)	461 (83.8)	0.342

LBW: Low birth weight; NBW: Normal birth weight’ p-value: Probability value.

^1^p-value is obtained from t-test for continuous and Chi-square test for categorical data.

### Risk factors of LBW using LR

The LR results of the different associated risk factors for LBW are presented in Table 3. Table 3 showed that Chittagong region, no educated mothers, the poorest and middle of wealth index, height, the child is twin, and child alive were significant risk factors of LBW (p<0.05). The LR result revealed that the mother from the Chittagong region is more likely to become LBW than the Barisal region (OR: 2.12, 95% CI: 1.15–3.88; p-value = 0.026). The babies whose mothers had no education experienced LBW 2.12 times (OR: 2.12, 95% CI: 1.15–3.88) higher than their higher educated mothers. The odds of having LBW babies were 2.09 times (OR: 2.09, 95% CI: 1.33–3.31; p-value: 002) higher for the poorest and 1.68 times higher for the middle wealth index than the richest. A single birth baby was less likely to be LBW than the second of multiple births (OR: 0.03, 95% CI: 0.01–0.07; p-value<0.001). The babies were more likely to become LBW compared to their counterparts if they were included: there was no child alive (OR: 0.03, 95% CI: 0.01–0.07; p-value: 0.001), and there was no delivery in CS (OR: 1.20, 95% CI: 0.94–1.54; p-value: 0.14).

**Table 3:** Identifying the risk factors of LBW using the LR.

Factors	OR	SE	p-value	95% CI of OR	
				Lower	Upper
**Region**					
Chittagong	1.75	0.44	0.03	1.07	2.88
Dhaka	1.17	0.30	0.54	0.71	1.94
Khulna	1.02	0.27	0.92	0.61	1.74
Mymensingh	0.69	0.20	0.20	0.39	1.21
Rajshahi	1.21	0.33	0.48	0.71	2.06
Rangpur	0.77	0.21	0.34	0.44	1.32
Sylhet	1.57	0.44	0.10	0.91	2.72
Barisal^®^	1.00				
**Education**					
No Education	2.12	0.66	0.02	1.15	3.88
Primary	1.27	0.25	0.23	0.87	1.86
Secondary	1.01	0.15	0.96	0.75	1.36
Higher^®^	1.00				
**Wealth**					
Poorest	2.09	0.49	<0.001	1.33	3.31
Poorer	1.19	0.27	0.43	0.77	1.84
Middle	1.68	0.31	0.01	1.17	2.42
Richer	1.28	0.23	0.16	0.91	1.82
Richest^®^	1.00				
**Height (m)**	0.02	0.02	<0.001	0.002	0.17
**Weight (kg)**	1.00	0.01	0.84	0.99	1.01
**Child is twin**					
Single birth	0.03	0.01	<0.001	0.01	0.07
1^st^ multiple birth	0.39	0.27	0.17	0.10	1.50
2^nd^ multiple birth^®^	1.00				
**Child is alive**					
No	2.71	0.75	<0.001	1.58	4.65
Yes^®^	1.00				
**Delivery in CS**					
No	1.20	0.15	0.14	0.94	1.54
Yes^®^	1.00				

®: Reference category; SE: Standard error; OR: Odds ratio; p-value: Probability value

### Comparisons between the performance of two different classifiers

DT and LR-based classifiers were adopted to predict LBW babies based on the significant risk factors (we get from LR). The entire data set is split into two sets as training set and test set using four different sets (Set-1: 70:30, Set-2: 75: 25, Set-3: 80:20, Set-4: 90: 10). Our findings show that the LR-based classifier was achieved an accuracy of 85.0% for Set-1, 85.3% for Set-2, 85.2% for Set-3, 87.6% for Set-4 (Table 4). So, Set-4 was the most accurate set for LR. Again, DT-based classifier showed an accuracy of 83.2% for Set-1, 82.5% for Set-2, 82.8% for Set-3, and 85.4% for Set-4 (Table 3). Therefore, LR and DT-based classifiers provided the highest accuracy for Set-4.

**Table 4:** Comparison of two classifier’s accuracy (%) over various holdout partitions.

Classifiers	Set-1	Set-2	Set-3	Set-4
**LR**	85.0	85.3	85.2	**87.6**
DT	83.2	82.5	82.8	85.4

The other performance evaluation parameters of two ML-based classifiers for set-4 were presented in Table 5. LR-based classifier shows Se (99.5%), Sp (17.7%), PPV (87.6%), NPV (85.7%), and AUC (0.59). Whereas, DT-based classifier gives Se (97.9%), Sp (11.8%), PPV (86.7%), NPV (50%), and AUC (0.55). In this performance assessment, the LR-based classifier was better than DT.

**Table 5:** Performance assessment (in %) of parameters for two ML-based classifiers.

Classifiers	Se	Sp	PPV	NPV	AUC
**LR**	**99.5**	**17.7**	**87.6**	**85.7**	**0.59**
DT	97.9	11.8	86.7	50	0.55

## Discussion

The current study determined the significant risk factors for LBW and predicted LBW babies using the critical risk factors with ML in Bangladesh. We implemented the LR-based method to determine the most significant risk factors. The LR method demonstrated that region, education, wealth, height, the child is twin, and the child is alive were the significant risk factors of LBW. Our study showed that the prevalence of LBW was 16.2% which was coincided with the prevalence of LBW (16.0%) (<2500 g) reported in BDHS, 2017–2018 [13]. Our findings showed that the prevalence of LBW varies from region to region. According to our study, the highest prevalence of LBW in Chittagong (20.8%) followed by Sylhet (20.1%), Rajshahi (16.1%), Barisal (15.6%), Dhaka (15.6%), Khulna (15.1%), Rangpur (13.8%) while the lowest prevalence of LBW was found in Mymensingh (11.3%). This was consistent with the report of BDHS, 2017–2018 [13]. It was noticed that the region was significantly associated with LBW. LR findings showed that Chittagong regions were more likely to be LBW babies than the Barisal region. For mothers with no education, the odds of having LBW babies were higher than mothers with higher education. A study conducted in Malawi showed that no educated mothers were at a higher risk of giving LBW babies [32]. Previous research had found that a mother’s education was a significant determinant of LBW [21, 23, 25, 40]. No educated mothers are not more aware of their health and nutrition than educated mothers. Bangladesh’s government should take the required steps to increase the literacy rate by incorporating a national education strategy and offering subsidies to women who live in the poorest families to attend school. Enhancing the education level will also diminish the prevalence of LBW.

Mothers from low-income families were more born babies with LBW than mothers from wealthy families. Low-income families cannot provide sufficient nutrition, medicine, and medical facilities to pregnant women, so they mostly give birth to LBW babies. Most of the families depend on only a single earning member due to lower family income. By being self-reliant, family members can contribute to the family income, which might help overcome poverty. The overall GDP of the country also postulates most families’ wealth condition. Increasing GDP can reduce poverty and LBW problems. Previous studies in developing countries, including India and Bangladesh, have shown the association between the wealth index and LBW [19, 25, 26, 40]. However, poverty and the absence of education (no education) were the most prominent risk factors associated with the prevalence of LBW babies in Bangladesh. LBW is more common in twin babies than in single birth babies. Mostly, second twin babies are at a higher risk of being LBW than single babies. Second-born twins may have an LBW because first-born twins have larger placental weights and more typically have a central insertion of the umbilical cord, both of which are positively connected with birth weight [41]. Lack of nutrition may cause death in the womb or after the birth of a baby. Babies who lack nutrition are born with LBW. Similar findings were found in earlier studies in Bangladesh and other developing countries [36–45]. In the present study, we employed two popular ML-based classifiers, namely LR and DT. We also used the hold-out cross-validation technique to measure the performance of the classifiers in terms of accuracy, Se, Sp, PPV, NPV, and AUC. Overall, performance assessment confirmed that the LR-LR-based combination is the superior combination for predicting LBW babies in Bangladesh.

## Strengths, limitations, and extension of the current study

The main contribution of this work is as follows: (i) Usage of nationally representative BDHS, 2017–18 dataset with large sample size; (ii) Utilization of sample weight that helped to achieve higher accuracy in the representing country; (iii) Utilization of t-test for continuous and Chi-square tests for categorical factors to show the association between different factors and LBW; (iv) Utilization of LR to detect the high-risk factors of LBW on the basis of OR and p-values (p-value<0.05); and (v) Utilization of two ML-based classifiers for the prediction of LBW babies. Although this study offered several contributions, it also had some limitations:

First, this study considered only limited factors. Second, the actual birth weight of babies was not reported in BDHS, 2017–18. We defined an LBW-based child size at birth. Third, we did not consider the gestational age of the mothers in our present study. Fourth, Only LR employs to extract the significant risk factors of LBW. Fourth: Usage of only two ML-based classifiers for the prediction of LBW babies.

We may use other feature selection methods like the random forest, principle component analysis, multilevel logistic regression, stepwise logistic regression, and so on instead of logistic regression. We may also adopt different classifiers like support vector machine, Gaussian process classification, artificial neural network, AdaBoost, and deep learning for the prediction of LBW. We also want to see the effect of LBW over time.

## Conclusion

LBW can be a leading cause of different diseases for babies at their adult age. It is sometimes dependent on key variables that we may manage or control. This study determined the informative risk factors and employed ML-based algorithms to predict LBW using the risk factors. However, mothers with an education are less likely to give birth to a child with LBW than no educated mother. Other factors like region, wealth index, mothers height, twin child, and alive child were significantly associated with LBW. Bangladesh’s government and health authorities should take the necessary steps to improve the economic condition of poor people and other significant factors in our country. This problem can be studied using ML-based algorithms. We believe that addressing the major factors that contribute to LBW will aid clinicians in predicting LBW before the child is born in Bangladesh.

### Recommendation

According to the current study, various demographic features are still significant for producing LBW in babies. It is possible to recommend that the government may create opportunities for women to access higher education and take a necessary step to improve the economic condition of poor people in Bangladesh.

## Supporting information

S1 Appendix (DOCX)
