# Supplementary material for: Predicting risks of low birth weight in Bangladesh with machine learning
Source: PLoS One. 2022 May 26;17(5):e0267190. doi: 10.1371/journal.pone.0267190 (PMC9135259; doi:10.1371/journal.pone.0267190)
Supplement: S1 Appendix — (DOCX) [file pone.0267190.s001.docx]

**Appendix**

Table A1. List of Abbreviations

| SN | Acronyms | Descriptions |
| --- | --- | --- |
| 1 | LBW | Low Birth Weight |
| 2 | ML | Machine Learning |
| 3 | LR | Logistic Regression |
| 4 | DT | Decision Tree |
| 5 | CV | Cross Validitation |
| 6 | AUC | Area Under the Curve |
| 7 | PPV | Positive Predictive Value |
| 8 | NPV | Negative Predictive Value |
| 9 | GRB | Gaussian Radial Basis |
| 10 | HT | Hyperbolic Tangent |
| 11 | CT | Classification tree |
| 12 | NB | Naïve Bayes |
| 13 | RF | Random forest |
| 14 | SVM | Support vector machine |
| 15 | NN | Neural Networks |
| 16 | RT | Random Tree |
| 17 | GCV | Generalized cross-validation |
| 18 | SMOTE | Synthetic Minority Oversampling Technique |
| 19 | K-NN | k-Nearest Neighbor |
